# Universal phonon mean free path spectra in crystalline semiconductors at high temperature

**DOI:** 10.1038/srep02963

**Published:** 2013-10-16

**Authors:** Justin P. Freedman, Jacob H. Leach, Edward A. Preble, Zlatko Sitar, Robert F. Davis, Jonathan A. Malen

**Affiliations:** 1 Department of Materials Science and Engineering, Carnegie Mellon University, Pittsburgh, PA, USA 15213; 2 Kyma Technologies, Raleigh, NC, USA 27617; 3 Department of Materials Science and Engineering, North Carolina State University, Raleigh, NC, USA 27695; 4 Department of Mechanical Engineering, Carnegie Mellon University, Pittsburgh, PA, USA 15213

## Abstract

Thermal conductivity in non-metallic crystalline materials results from cumulative contributions of phonons that have a broad range of mean free paths. Here we use high frequency surface temperature modulation that generates non-diffusive phonon transport to probe the phonon mean free path spectra of GaAs, GaN, AlN, and 4H-SiC at temperatures near 80 K, 150 K, 300 K, and 400 K. We find that phonons with MFPs greater than 230 ± 120 nm, 1000 ± 200 nm, 2500 ± 800 nm, and 4200 ± 850 nm contribute 50% of the bulk thermal conductivity of GaAs, GaN, AlN, and 4H-SiC near room temperature. By non-dimensionalizing the data based on Umklapp scattering rates of phonons, we identified a universal phonon mean free path spectrum in small unit cell crystalline semiconductors at high temperature.

The physics of heat transport in condensed matter is critical to thermal management in a diverse range of technologies as well as thermoelectric energy conversion. The dominant carriers of heat in non-metallic crystalline materials are phonons, defined as quantized lattice vibrations. Specific heat, phonon group velocity, and phonon mean free path (MFP)–the average distance a phonon travels between scattering events–determine a material's thermal conductivity. Recent studies have shown that it is possible to experimentally measure the MFP dependent contributions of phonons to thermal conductivity–the phonon MFP spectrum^1^,^2^,^3^,^4^,^5^. In this work we present experimental evidence of a universal phonon MFP spectrum in several crystalline semiconductors. Broadband frequency domain thermoreflectance (BB-FDTR)^1^, an optical pump-probe technique, was used to measure the integrated phonon MFP spectrum, referred to as the thermal conductivity accumulation function (*k*_accum_) of single crystal (100) gallium arsenide (GaAs), (0001) gallium nitride (GaN), (0001) aluminum nitride (AlN), and (0001) 4H-silicon carbide (SiC) at temperatures (*T*) near 80 K, 150 K, 300 K, and 400 K. By non-dimensionalizing the data based on Umklapp scattering rates of phonons, which is the dominant resistive scattering mechanism at high temperature (herein meaning high relative to the temperature of peak thermal conductivity), we discovered a universal thermal conductivity accumulation function (*k*_universal_).

GaAs, GaN, AlN, and SiC are critical to the high power electronics and optoelectronics industries^6^. Primary applications of these materials include high electron mobility transistors^7^, multi-junction solar cells^8^, and light emitting diodes^9^,^10^, where poor heat dissipation leads to electrical and optical inefficiencies and shorter lifetimes^11^. The bulk thermal conductivities of GaAs, GaN, AlN, and SiC (4H and 6H) at *T* = 300 K are 50^12^,^13^, 230^14^,^15^, 285^16^, and 490 Wm^−1^K^−1^^17^,^18^, respectively. First principles calculations have been used to evaluate *k*_accum_ of GaAs, though the predictions have not been experimentally confirmed^19^. Thin film architectures and nanostructures possess reduced thermal conductivities compared to bulk materials due to boundary scattering, which limits phonon MFPs^20^,^21^,^22^. Heat sources with dimensions smaller than phonon MFPs also perceive a locally suppressed thermal conductivity^3^,^23^. Therefore, experimental measurements of the intrinsic *k*_accum_ are required to understand these effects, which are prevalent in GaAs, GaN, AlN, and SiC technologies.

Kinetic theory can be used to derive an approximate expression for thermal conductivity, *k*, as 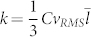, where *C* is the volumetric heat capacity, *v_RMS_* is the root-mean-squared velocity, and 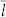 is the average MFP that a particle travels between scattering events. Though this gray approximation for the MFP of particles provides accurate predictions of thermal conductivity in gases, it does not accurately predict thermal conductivity in solids, where phonons exhibit a broad distribution of MFPs^24^. To accurately describe thermal conductivity in a non-metallic crystal, a summation weighted by the mode-dependent phonon properties is required. While the heat capacity and phonon group velocity can be predicted and measured, experimental determination of the phonon MFP spectrum has remained a challenge. To resolve thermal conductivity as a function of phonon MFP, the thermal conductivity accumulation function was defined as^24^, 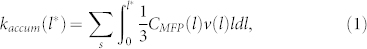
where *l* is the phonon MFP, *v* is the phonon group velocity, *C*_MFP_ is the volumetric heat capacity per unit phonon MFP, and *s* indexes the polarization of phonons. Since the integral is taken from 0 to l*, *k*_accum_ quantifies the contribution to bulk thermal conductivity of phonons with a MFP less than or equal to l*.

Experimental measurements of *k*_accum_ have been reported using BB-FDTR, time domain thermoreflectance (TDTR), and transient grating techniques^1^,^2^,^3^,^4^,^25^. Based on observations of suppressed thermal conductivity in semiconductor alloys, Koh and Cahill^2^ hypothesized that the thermal penetration depth, 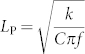, limited the diffusive phonons interrogated by TDTR to those having a MFP less than *L*_P_, where *f* was the modulation frequency of the pump laser. Further studies using TDTR have equated l* to the laser spot size on Si^3^ and the dimensions of nano-patterned heaters on sapphire^26^. More recently, transient grating experiments have varied the grating period to study related ballistic phonon transport effects in Si membranes^4^.

The BB-FDTR apparatus used to measure *k*_accum_ is shown in Fig. 1(a). The wavelengths of the continuous wave pump and probe lasers in BB-FDTR are 488 nm and 532 nm, respectively. The pump laser is intensity modulated by an electro-optic modulator (EOM) at a frequency *f*_1_, which upon absorption in the Au transducer film induces a temperature response in the sample at frequency *f*_1_. The continuous wave probe laser beam then reflects off the sample with a modulated intensity at frequency *f*_1_, as a result of Au's thermoreflectance. The reflected pump and probe have identical frequencies with a phase difference representing the phase-lag of temperature to heat flux at the sample surface. The pump and probe signals are then heterodyned by a second EOM at frequency *f*_2_ to reduce coherent and ambient noise as described in the Supplementary Information. To obtain a plot of accumulated thermal conductivity as a function of phonon MFP, phase-lag data from BB-FDTR was fit to an analytical solution of the heat diffusion equation in a layered medium^27^.

BB-FDTR and TDTR measurements have demonstrated that a constant value of thermal conductivity over the range of measured heating frequencies (0.2 ≤ *f_1_* ≤ 200 MHz) accurately identifies the expected bulk values of SiO_2_ and platinum samples with little deviation between the measured data and analytical fits^1^,^2^. This result suggests that a constant value of thermal conductivity is appropriate when the MFPs of energy carriers are much shorter than *L*_P_. On the other hand, a constant thermal conductivity over all heating frequencies was found to under predict thermal conductivity in single crystal Si^1^, and likewise in the GaAs, GaN, AlN, and 4H-SiC crystals studied here. As an example, the measured data and constant thermal conductivity fit, shown in Fig. 1(b), yield a bulk thermal conductivity value of 194 Wm^−1^K^−1^ for 4H-SiC at *T* = 304 K (40% of *k*_bulk_). This suppression is expected when *L*_P_ is shorter than the MFPs of some phonons^1^,^2^. Phase-lag data was instead divided into overlapping windows of 13 data points and the thermal conductivity of each window was individually fit. The thermal conductivity of 4H-SiC extracted from each window, *k*_j_, is plotted in Fig. 1(c) as a function of the windows' median frequency and decreases from 294 Wm^−1^K^−1^ to 74 Wm^−1^K^−1^ over the frequency range (200 kHz to 200 MHz).

To generate an accumulation function, the thermal conductivity was plotted against 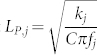, where *f*_j_ was the median frequency of the j^th^ window. At low *f*, phonon transport was primarily diffusive, while at high *f*, phonons with a MFP > *L*_P_ traveled ballistically as illustrated in Fig. 1(d). Consistent with prior studies, we assumed that ballistic phonons did not contribute to the measured value of thermal conductivity^1^,^2^,^3^,^4^,^26^. This convention equates l* to *L*_P_, and provides a reasonable comparison between experimental *k*_accum_ and theoretical predictions^1^,^2^, though it has not been rigorously proven and neglects spot size effects that have influenced TDTR measurements in Si^3^.

## Results

**Figure 2:**
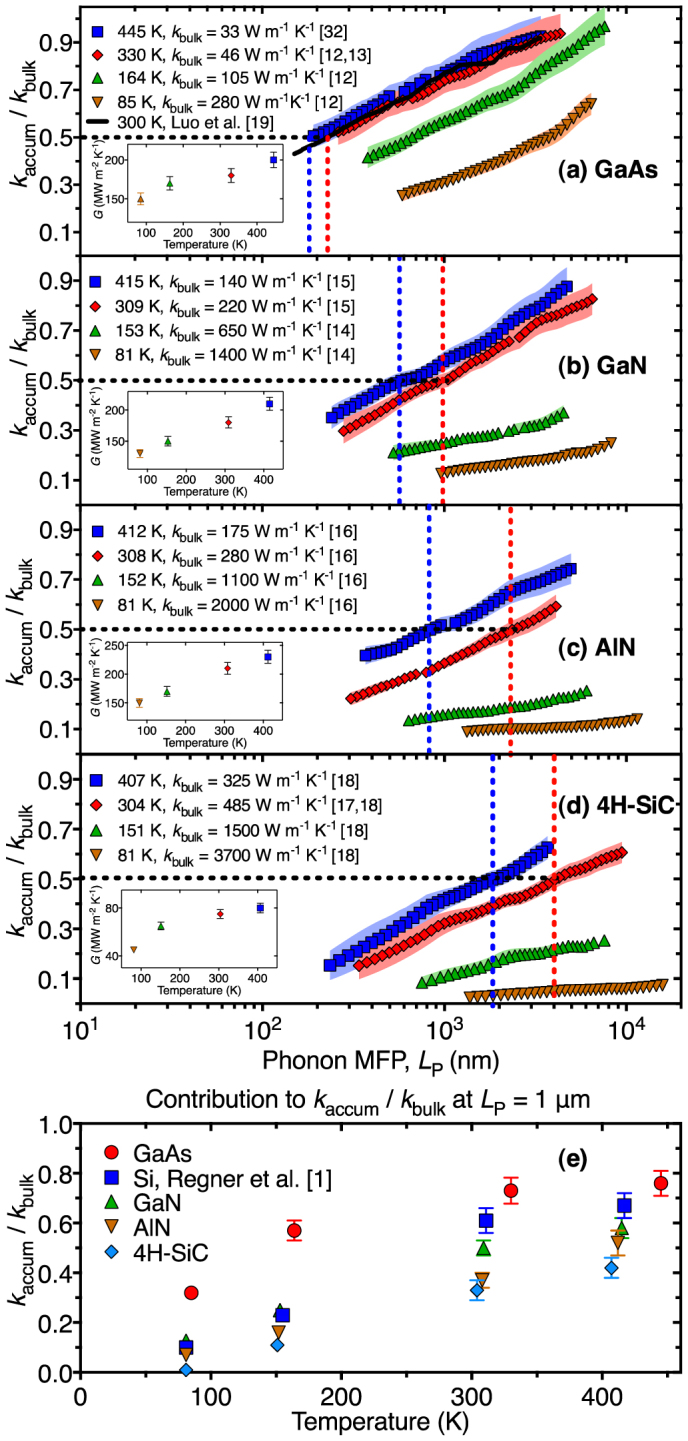
(a)–(d) Normalized thermal conductivity accumulation functions of GaAs, GaN, AlN, and 4H-SiC at temperatures near 80 K, 150 K, 300 K, and 400 K as a function of phonon MFP or *L*_P_. Temperatures are offset from these nominal values due to laser heating (see Supplementary Information). Inset are the thermal interface conductance values, *G*, between the substrate and the gold-chromium transducer layer. (e) The contribution to thermal conductivity of phonons with a MFP < 1 μm at temperatures near 80 K, 150 K, 300 K, and 400 K.

The *k*_accum_ of GaAs, GaN, AlN, and 4H-SiC at temperatures near 80 K, 150 K, 300 K, and 400 K are displayed in Fig. 2 (offset temperatures due to laser heating are added to the nominal temperatures measured at the cryostat cold finger). Insets report the measured thermal interface conductance at each temperature. For all four materials the thermal interface conductance, *G*, increased with temperature in agreement with prior studies of metal-dielectric interfaces^28^. Interfaces of AlN and GaN with chromium have been measured at *T* = 300 K and our results are within 20% of the values^29^. The *k*_accum_ data is normalized by the bulk thermal conductivity at each temperature. Bulk values of thermal conductivity are an additional source of uncertainty that are not reflected in the error bars on Fig. 2 (see Supplementary Information for justification of the chosen bulk values). Density functional theory driven simulations of *k*_accum_ in GaAs at *T* = 300 K agree with the measured data in Fig. 2(a)^19^. BB-FDTR measurements indicate that phonons with MFPs less than 230 ± 120 nm contribute 50% to the total bulk thermal conductivity of GaAs at *T* = 330 K. As the bulk thermal conductivity of the material increases, phonons with longer MFPs contribute more significantly to the bulk thermal conductivity. Phonons with MFPs less than 1000 ± 200 nm, 2500 ± 800 nm, and 4200 ± 850 nm contribute 50% to the bulk thermal conductivity of GaN, AlN, and 4H-SiC at *T* = 309 K, 308 K, and 304 K, respectively.

An isotropic model of thermal conductivity, where *k_in-plane_* = *k_cross-plane_*, was assumed in GaAs, GaN, AlN, and 4H-SiC across all heating frequencies. This assumption is valid for GaAs as its cubic (zinc-blende) crystal structure contains high-order symmetry and its thermal conductivity tensor is isotropic^30^. Slack et al.^16^ reason that in-plane and cross-plane thermal conductivities of AlN varied by less than 5% at room temperature and above. GaN was assumed to exhibit similar quasi-isotropic behavior to that of AlN due to the fact that GaN and AlN have identical lattice structures and similar acoustic wave velocity deviation between their in-plane and cross-plane velocities^31^,^32^. Finally, the thermal conductivity of 6H-SiC and 4H-SiC along the [0001] axis has been found to be about 20–30% less than the thermal conductivity along the [1000] axis at room temperature^17^,^33^. Since *L*_P_ ~ *r* (*r* = 2.65 ± 0.13 μm, 1/*e*^2^ radius laser spot) for much of the frequency range in 4H-SiC, heat spreading is not purely one-dimensional. Yet the data must be normalized to a single value. Therefore, we have chosen the [1000] axis bulk values because they are reported over a wide range of temperatures. To examine the effects of anisotropy in 4H-SiC, a heat diffusion model considering in-plane and cross-plane thermal conductivities separately was used to fit phase-lag data as a function of heating frequency^34^, where *k_cross-plane_* = 0.8*k_in-plane_* was held constant. The observed thermal conductivity difference between the anisotropic and isotropic data interpretations varied by less than 11% of *k*_bulk_ at *T* = 407 K and 304 K and by less than 4% of *k*_bulk_ at *T* = 151 K and 81 K (see Supplementary Information). Therefore, data presented in Fig. 2 assumed isotropic thermal conductivity in GaAs, GaN, AlN, and 4H-SiC.

Figure 2(e) compares the contribution of phonons with a MFP < 1 μm to the bulk thermal conductivity in GaAs, Si^1^, GaN, AlN, and 4H-SiC from 81 K to 445 K. As the bulk thermal conductivity of the material increases, phonons with a MFP < 1 μm contribute less to the total thermal conductivity. As the temperature decreases, phonon occupation and phonon-phonon scattering are reduced, and long MFP phonons (>1 μm) become the dominant heat carriers. With the approaches from Ref. 35 the data in Fig. 2 allows for mapping of thermal conductivity suppression in nanostructured materials and devices.

## Discussion

Using measured *k*_accum_ data a universal thermal conductivity accumulation function was identified. Umklapp scattering is the dominant resistive scattering process above the temperature of peak thermal conductivity, which typically occurs at less than 10% of the Debye temperature in bulk crystalline materials with low impurity concentration^36^. To derive an expression for *k*_accum_ at high temperatures, the following form for the relaxation time, *τ*, of phonon scattering due to Umklapp processes was assumed^35^, 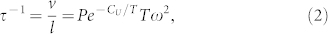
where *P* and *C*_U_ are material dependent constants that describe Umklapp scattering rates, and *ω* is the phonon frequency. We employed the truncated Debye dispersion^21^ *ω* = *v_s_q* for acoustic phonons, where *v*_s_ is the sound velocity and *q* is the phonon wave vector. The Debye dispersion is truncated at the Brillouin zone edge frequency, *ω_BZE_* of the real dispersion relationship as not to overestimate contributions from high frequency, low group velocity acoustic phonons. An isotropic expression for *k*_accum_ follows from Eq. (1) for a single polarization^24^, 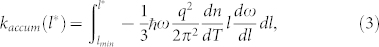
where 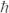 is the reduced Planck constant, *n* is the Bose-Einstein distribution, and 
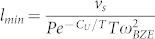
 is the minimum phonon MFP determined by *ω_BZE_*. Classical occupation leads to 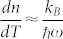, where *k*_B_ is the Boltzmann constant. This assumption is accurate for phonons having 
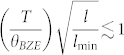
, where 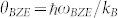, as derived in section 5 of the Supplementary Information. Since thermal conductivity in intrinsic crystalline semiconductors results largely from phonons with 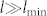, this assumption can be valid even when *T* < *θ_BZE_*, as it is for our materials. Therefore, the normalized *k*_accum_, including acoustic longitudinal and transverse modes is (see Supplementary Information for a full derivation and tabulated values of *P*, *C*_U_, *v*_s_, and *ω_BZE_*), 
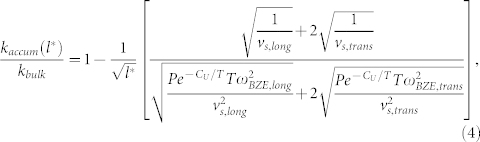
Based upon inspection of Eq. (4) a non-dimensional phonon MFP is identified as, 
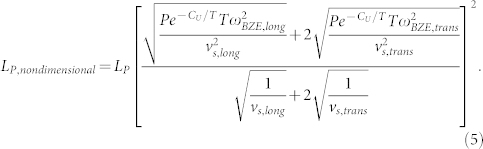
The assumption of classical occupation is neccesary for deriving Eqn. (4). The form of *L*_p,nondimensional_ (Eqn. (5)) identified in this derivation is valid, independant of occupation, when Umklapp scattering dominates thermal resistance.

**Figure 3:**
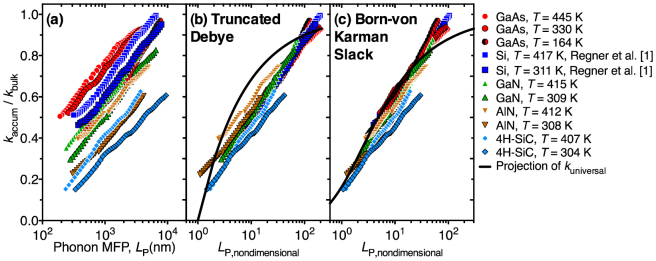
(a) Normalized thermal conductivity accumulation functions of GaAs, Si^1^, GaN, AlN, and 4H-SiC as a function of phonon MFP or *L*_P_. (b) Normalized thermal conductivity accumulation functions as a function of *L*_P,nondimensional_ (Eq. (5)) based on the truncated Debye model in GaAs, Si, GaN, AlN, and 4H-SiC. The *k*_accum_ data collapse to a universal thermal conductivity accumulation function. (c) Normalized thermal conductivity accumulation functions as a function of *L*_P,nondimensional_ (Eq. (6)) based on the Born-von Karman Slack model in GaAs, Si, GaN, AlN, and 4H-SiC. The *k*_accum_ data collapse to a universal thermal conductivity accumulation function. Solid lines in (b) and (c) show the projection of *k*_universal_ from truncated Debye and Born-von Karman Slack models.

The assumption of Umklapp dominated scattering is adequately met by Si, GaN, AlN, and 4H-SiC at *T* ≈ 300 K and 400 K and GaAs at *T* ≈ 150 K, 300 K, and 400 K given that the temperatures of their peak thermal conductivity occur at ~ 25 K^37^, ~ 55 K^14^, ~ 60 K^16^, ~ 50 K^18^, ~ 20 K^12^, respectively. The *k*_accum_ of GaAs, Si^1^, GaN, AlN, and 4H-SiC, as normalized by *k*_bulk_, are shown in Fig. 3(a). When plotted as a function of *L*_P,nondimensional_, as in Fig. 3(b), *k*_accum_ data collapse to a single universal thermal conductivity accumulation function, *k*_universal_. This collapse equivalently implies a universal phonon MFP spectrum in these materials, as the accumulation function is the integral of the MFP spectrum. To test the robustness of *k*_universal_ the Born-von Karman Slack model^24^,^35^ was also used to find values of *P* and *C*_U_, where an average sound velocity, 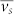, and Brillouin zone edge frequency was used for the longitudinal and transverse branches. In this case, *L*_P,nondimensional_ is defined as, 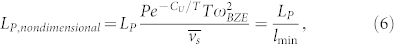
Fig. 3(c) shows that a *k*_universal_ based on the Born-von Karman Slack model also exists when using the form of *L*_P,nondimensional_ in Eq. (6). The scale of the *L*_P,nondimensional_-axis in Fig. 3(b) and Fig. 3(c) shows that approximately 90% of thermal conductivity in crystalline semiconductors results from phonons with MFPs 1–200 times the MFP of Brillouin zone edge acoustic phonons (*l*_min_). For comparison the projections of *k*_universal_ based on truncated Debye and Born-von Karman Slack models are shown in Figure 3b and 3c.

Some debate exists on the importance of isotope scattering in gallium-based semiconductors, though authors agree that it is diminished at high temperatures^31^,^38^,^39^,^40^. The collapse of GaAs and GaN data to *k*_universal_ indicates that isotope scattering did not strongly affect the *τ*^−1^ ∝ *ω*^2^ scattering of long MFP phonons probed by BB-FDTR.

Knowledge of *k*_accum_ enables mapping of thermal conductivity suppression in nanostructures and devices based on their characteristic size and is therefore critical to the thermal design of electronic, photonic, and thermoelectric technologies. The existence of a material independent *k*_universal_ in GaAs, Si, GaN, AlN, and SiC suggests that the phonon mean free path spectrum is a universal feature of intrinsic crystalline semiconductors. This is useful for the projection of *k*_accum_ in intrinsic crystalline semiconductors where thermal resistance is dominated by Umklapp processes based on parameters that can be obtained from historical thermal conductivity vs. temperature data. Because our data is based on small unit cell, high thermal conductivity materials, additional experiments are needed to determine whether more complex materials such as alloys and large unit cell crystals adhere to a different form of *k*_univeral_.

## Methods

### Sample preparation

Undoped, semiconductor grade (100) GaAs, (0001) GaN, (0001) AlN, and (0001) 4H-SiC bulk wafer samples were purchased and provided by University Wafer Inc., Kyma Technologies Inc., HexaTech Inc., and Cree Inc., respectively. Preparation of bulk wafer samples for BB-FDTR measurements included two steps. 4H-SiC was dipped into an HF:H_2_0 (1:9) solution for 10 minutes at room temperature to remove the native oxide layer and blown dry in ultra high purity nitrogen. Next, a chromium adhesion layer and a gold transducer layer of thickness reported in Supplementary Information Table S1 were deposited on GaAs, GaN, AlN, and 4H-SiC samples via a Perkin Elmer 6 J sputtering system. Gold was chosen as the transducer material, as it has a high absorptivity at 488 nm (pump laser) and a high coefficient of thermoreflectance at 532 nm (probe laser)^41^. X-ray reflectivity measurements were used to determine gold and chromium layer thicknesses, as detailed in Supplementary Information section S2.

### Broadband frequency domain thermoreflectance

To measure a large range of *k*_accum_, BB-FDTR heterodynes the pump and probe signals present at *f_1_* with a second modulation at frequency *f*_2_ producing signals at *f*_2_ − *f*_1_ and *f*_1_ + *f*_2_. This allows for heating frequencies up to 200 MHz^25^. This was highly advantageous compared to traditional frequency domain thermoreflectance (FDTR), where the signal is compromised by coherent and ambient noise at frequencies greater than 20 MHz. The component of the signal with frequency *f*_1_ + *f*_2_ is filtered out and the lower frequency component, *f*_2_ − *f*_1_, is recorded using an SR-830 lock-in amplifier. An optical band pass filter is used to attenuate the pump or probe beam to permit for measurement of each beam individually. Frequencies *f*_1_ and *f*_2_ are concurrently swept between 200 kHz and 200 MHz while maintaining *f*_2_ − *f*_1_ = 86 kHz. A constant 1/e^2^ spot size radius of 2.65 ± 0.13 μm, measured using a knife-edge technique, was used for all data presented.

To fit thermal conductivity and thermal interface conductance two assumptions were made. (1) The thermal interface conductance between the sample and the chromium-gold layer was frequency independent. This was reasonable as the heating frequency was five orders of magnitude smaller than the relaxation rate of electrons in the gold layer at room temperature^42^. Hence, at the interface the electrons and phonons will be in equilibrium, independent of modulation frequency. (2) The volumetric heat capacity was frequency independent. This assumption is valid, as the long MFP phonons that dominate thermal conductivity do not contribute significantly to the heat capacity of a semiconducting material^1^,^43^.

## Author Contributions

J.P.F. prepared samples and performed all measurements. J.H.L., E.A.P., Z.S. and R.F.D. provided samples. J.P.F. and J.A.M. wrote the manuscript. All authors discussed the data and edited the manuscript.

## Supplementary Material

Supplementary Information Supplementary Information

## Figures and Tables

**Figure 1:**
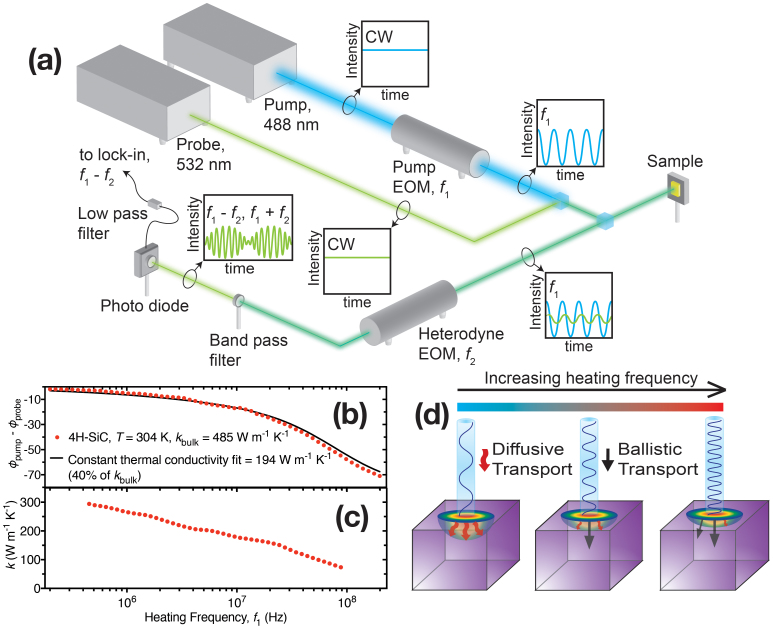
(a) BB-FDTR experimental apparatus. (b) Thermal response of the probe phase (temperature) subtracted from the pump phase (heat flux). Fitting data with an analytical solution to the thermal diffusion equation that assumes a single value of thermal conductivity over all frequencies yields a poor fit and an under prediction of *k*_bulk_. (c) Window fitting over small frequency ranges yields the thermal conductivity as a function of the window's median frequency. (d) The pump laser induces a periodic heat flux that causes a thermal response with a characteristic penetration depth. The greater the heating frequency, the smaller the thermal penetration depth is, causing fewer diffusive phonons to contribute to the measured thermal conductivity.
