# Supplementary material for: Universal phonon mean free path spectra in crystalline semiconductors at high temperature
Source: Sci Rep. 2013 Oct 16;3:2963. doi: 10.1038/srep02963 (PMC3797423; doi:10.1038/srep02963)
Supplement: Supplementary Information [file srep02963-s1.pdf]

*Supplementary Information for "**Universal phonon mean free path spectra in crystalline semiconductors at high temperature**"*

Justin P. Freedman, Jacob H. Leach, Edward A. Preble, Zlatko Sitar, Robert F. Davis, and Jonathan A. Malen

1. X-Ray Reflectivity (XRR) – thickness of gold and chromium layers

An example of an x-ray reflectivity plot yielding a 7 nm chromium film and a 58 nm gold film on top of a gallium arsenide substrate is shown in Figure S1. The green and blue plots show the sensitivity of the fit (± 2 nm of Au). Curves are offset in the y-position from the XRR data for viewing purposes, but clear variations are present in the positions and shapes of the humps. XRR data is sensitive to three fitting parameters: film thickness, roughness, and density. The analysis assumed a bulk value of density, while the thickness and roughness of the films were varied.

Figure S1:

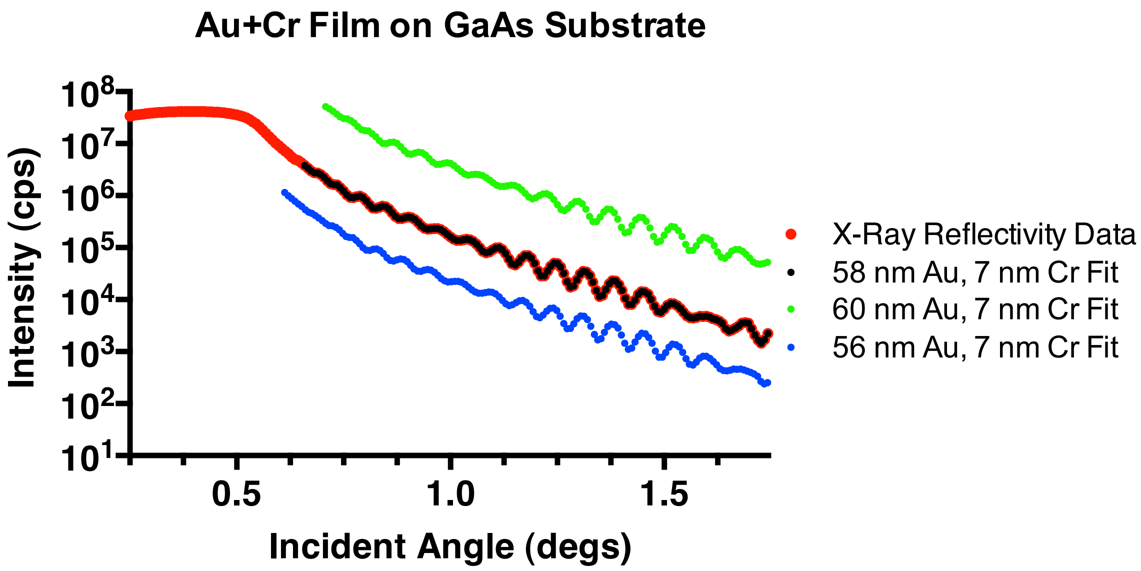

2. Parameters used to fit phase lag data

Two unknown fitting parameters were extracted by fitting the experimental phase vs. frequency data: the thermal conductivity of the sample and the thermal interface conductance between the sample and the gold-chromium transducer layer. The value of thermal interface conductance was fit by minimizing the mean square error of the analytical model in the high

frequency range of beam modulation, where the signal is most sensitive to its value. Uncertainty in k_{accum} was determined based on the uncertainty in the input parameters to the fitting program, as described in Ref. [1-3].

T = Temperature of cryostat cold finger [K]

C = Volumetric heat capacity [J cm^{-3} K^{-1}]

G = Interface thermal conductance [MW m^{-2} K^{-1}]

L = Thickness of gold or chromium film [nm] (Measured by XRR – see S7)

r = 1/e^2 spot size radius [µm] (Measured by knife-edge technique)

A = Absorbed laser power [mW]

ΔT$_{DC}$ = DC temperature rise [K], based on $\Delta T_{DC} = A/(\sqrt{\pi}\,2rk)$ [4], where k [W m^{-1} K^{-1}] is the bulk thermal conductivity of the substrate

Table S1(a): GaAs

T	C_{Cr}	C_{Au}	C_{GaAs} [5]	$G_{Au-GaAs}$	L_{Cr}	L_{Au}	r	A	ΔT_{DC}
80	1.44	1.88	0.75	150±12	7±1	58±2	2.65±0.13	14±1	4.5
150	2.16	2.26	1.22	175±14	7	58	2.65	14	13.6
300	3.23	2.47	1.76	180±14	7	58	2.65	14	29.8
400	3.49	2.47	1.76	200±16	7	58	2.65	14	45.2

Table S1(b): GaN

T	C_{Cr}	C_{Au}	C_{GaN} [6]	G_{Au-GaN}	L_{Cr}	L_{Au}	r	A	ΔT_{DC}
80	1.44	1.88	0.62	130±10	7±1	56±2	2.65±0.13	20±1	1.4
150	2.16	2.26	1.60	150±12	7	56	2.65	20	3.1
300	3.23	2.47	3.01	180±14	7	56	2.65	20	9.3
400	3.49	2.47	3.44	210±17	7	56	2.65	20	14.7

Table S1(c): AlN

T	C_{Cr}	C_{Au}	C_{AlN} [6]	G_{Au-AlN}	L_{Cr}	L_{Au}	r	A	ΔT_{DC}
80	1.44	1.88	0.33	150±12	4±1	52±2	2.65±0.13	20±1	1.1

150	2.16	2.26	1.17	170±14	4	52	2.65	20	2.0
300	3.23	2.47	2.41	210±17	4	52	2.65	20	7.5
400	3.49	2.47	2.93	230±18	4	52	2.65	20	11.9

Table S1(d): 4H-SiC

**T**	**C_{Cr}**	**C_{Au}**	**C_{SiC}** [6]	**$G_{Au\text{-}SiC}$**	**L_{Cr}**	**L_{Au}**	**r**	**A**	**ΔT_{DC}**
80	1.44	1.88	0.16	45±4	6±1	56±2	2.65±0.13	20±1	0.6
150	2.16	2.26	0.80	65±5	6	56	2.65	20	1.2
300	3.23	2.47	2.31	75±6	6	56	2.65	20	4.4
400	3.49	2.47	2.89	80±7	6	56	2.65	20	6.9

3. Phase data as a function of heating frequency

Below is a figure containing the phase-lag data for each material and temperature along with the solution to the heat diffusion equation assuming a constant thermal conductivity across all measured heating frequencies. A constant thermal conductivity does not fit the phase-lag data and under predicts the bulk thermal conductivity of each material. Fits are based on parameters from Table S1(a)-(d).

Figure S2:

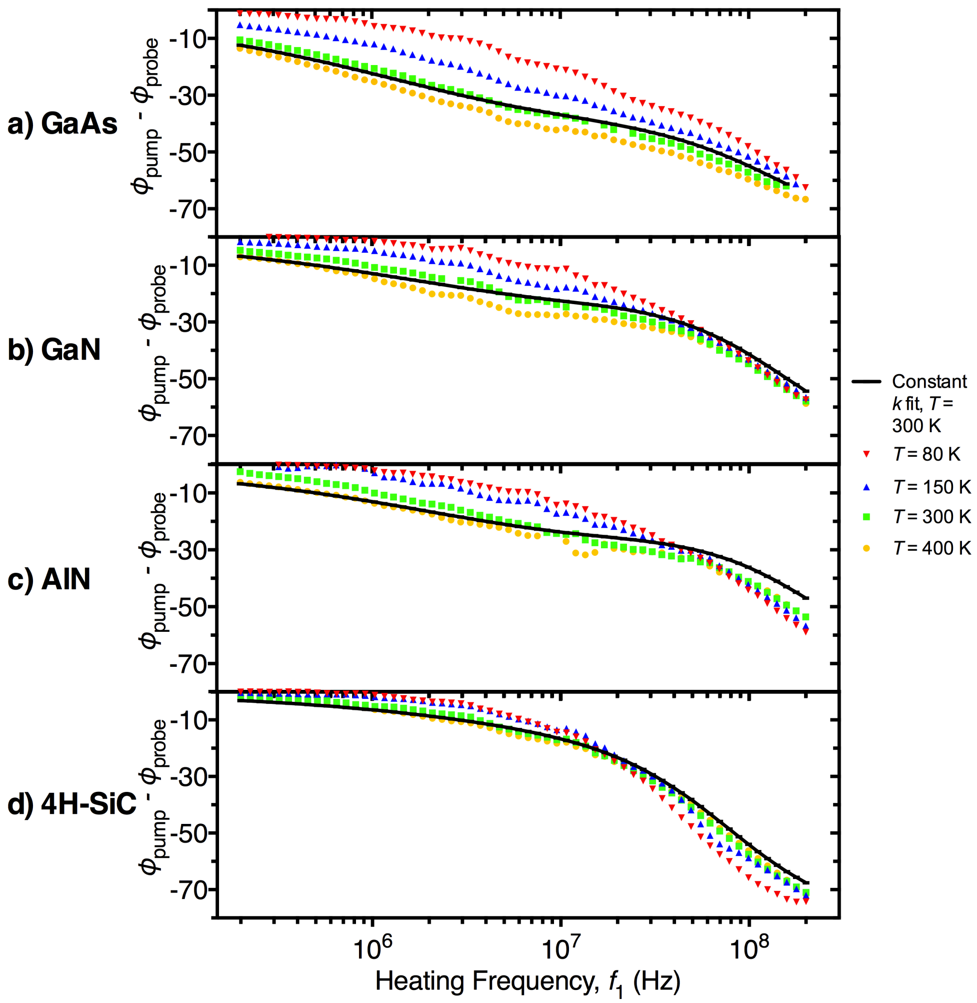

4. Derivation of universal phonon MFP normalization factor

The following is a step-by-step derivation of the thermal conductivity accumulation function as a function of phonon mean free path, where Umklapp scattering is assumed to be the dominant thermal resistance mechanism. The universal phonon MFP normalization factor results from the derivation. To begin, we assume Umklapp scattering given by the form in Ref. [7] as the dominant phonon scattering mechanism

$$\tau_{Umklapp}^{-1} = Pe^{-C_U/T}T\omega^2 \qquad \text{S1}$$

τ = Relaxation time of phonon scattering

P and C_U = Material dependent constants that determine rates of Umklapp scattering

ω = Phonon frequency

T = Temperature

v = Phonon group velocity

l = Phonon mean free path

Using $v = v_s$, where v_s is the velocity of sound in the material,

$$\omega = \sqrt{\frac{v_s}{Pe^{-C_U/T}Tl}} \qquad \text{S2}$$

so,

$$\frac{\partial \omega}{\partial l} = -\frac{1}{2}\left(\frac{1}{l}\right)^{3/2}\sqrt{\frac{v_s}{Pe^{-C_U/T}T}} \qquad \text{S3}$$

and

$$q = \frac{\omega}{v_s} = \sqrt{\frac{1}{Pe^{-C_U/T}Tv_s l}} \qquad \text{S4}$$

q = Wave vector

Now, using the thermal conductivity accumulation function for a single polarization,

$$k_{accum}\left(l^*\right) = \int_0^{l^*} k_l\, dl = \int_{l_D}^{l^*} -\frac{1}{3}\hbar\omega \frac{q^2}{2\pi^2}\frac{\partial n}{\partial T} l \frac{\partial \omega}{\partial l} dl \quad \text{S5}$$

k = Thermal conductivity

$\hbar$ = Reduced Planck constant

n = Bose-Einstein distribution

Substituting the equations above into the thermal conductivity accumulation function from Eqn. S5, one finds

$$k_{accum}\left(l^*\right) = \int_0^{l^*} \frac{\hbar}{12\pi^2}\left(\frac{1}{Pe^{-C_U/T}Tl}\right)^2 \frac{\partial n}{\partial T} dl \quad \text{S6}$$

Classical occupation of the long MFP phonons probed by BB-FDTR leads to,

$$\frac{\partial n}{\partial T} \approx \frac{k_B}{\hbar\omega} = \frac{k_B}{\hbar}\sqrt{\frac{Pe^{-C_U/T}Tl}{v_s}} \qquad \text{S7}$$

The validity of this approximation is discussed in Section 5 of the supplementary information. Given the truncated Debye approximation, the lower limit on the integral is no longer zero. It is determined by the Brillouin zone edge frequency, ω_{BZE}, as

$$l_{min} = \frac{v_s}{Pe^{-C_U/T}T\omega_{BZE}^2} \quad \text{S8}$$

such that the un-normalized expression for k_{accum} for one polarization is,

$$k_{accum}\left(l^*\right) = \int_{l_{min}}^{l^*} \frac{k_B}{12\pi^2\sqrt{v_s}}\left(\frac{1}{Pe^{-C_U/T}Tl}\right)^{3/2} dl \quad \text{S9}$$

Now, when k_{accum} is normalized by the bulk value and both longitudinal and transverse modes are considered, where $k = k_{long} + 2k_{trans}$,

$$\frac{k_{accum}\left(l^*\right)}{k_{bulk}} = \frac{\int_{l_{min,long}}^{l^*} \frac{k_B}{12\pi^2\sqrt{v_{s,long}}}\left(\frac{1}{Pe^{-C_U/T}Tl_{long}}\right)^{3/2} dl_{long} + 2\int_{l_{min,trans}}^{l^*} \frac{k_B}{12\pi^2\sqrt{v_{s,trans}}}\left(\frac{1}{Pe^{-C_U/T}Tl_{trans}}\right)^{3/2} dl_{trans}}{\int_{l_{min,long}}^{\infty} \frac{k_B}{12\pi^2\sqrt{v_{s,long}}}\left(\frac{1}{Pe^{-C_U/T}Tl_{long}}\right)^{3/2} dl_{long} + 2\int_{l_{min,trans}}^{\infty} \frac{k_B}{12\pi^2\sqrt{v_{s,trans}}}\left(\frac{1}{Pe^{-C_U/T}Tl_{trans}}\right)^{3/2} dl_{trans}} \quad \text{S10}$$

which leads to an expression for k_{accum}, where P and C_U are assumed to be independent of polarization (assumed for simplicity during our fitting of k vs. T data),

$$\frac{k_{accum}\left(l^*\right)}{k_{bulk}} = 1 - \frac{1}{\sqrt{l^*}}\left[\frac{\sqrt{\frac{1}{v_{s,long}}} + 2\sqrt{\frac{1}{v_{s,trans}}}}{\sqrt{\frac{Pe^{-C_U/T}T\omega_{BZE,long}^2}{v_{s,long}^2}} + 2\sqrt{\frac{Pe^{-C_U/T}T\omega_{BZE,trans}^2}{v_{s,trans}^2}}}\right] \quad \text{S11}$$

Therefore, the non-dimensional phonon MFP was defined as,

$$L_{P,nondimensional} = L_P\left[\frac{\sqrt{\frac{Pe^{-C_U/T}T\omega_{BZE,long}^2}{v_{s,long}^2}} + 2\sqrt{\frac{Pe^{-C_U/T}T\omega_{BZE,trans}^2}{v_{s,trans}^2}}}{\sqrt{\frac{1}{v_{s,long}}} + 2\sqrt{\frac{1}{v_{s,trans}}}}\right]^2 \quad \text{S12}$$

When the Umklapp relaxation times were found using the Born-von Karman Slack model, as opposed to the truncated Debye model, an average sound velocity, $\overline{v_s}$, and Brillouin zone edge frequency were used. When this assumption is made, Eqn. S12 simplifies to,

$$L_{P,nondimensional} = L_P \frac{Pe^{-C_U/T}T\omega_{BZE}^2}{\overline{v_s}} \quad \text{S13}$$

Leading us to define the non-dimensional phonon MFP described in Eqn. S13.

5. Validity of the high temperature approximation for ∂n/∂T

Here we compare the exact expression for $\partial n/\partial T$ with the high temperature approximation $k_B/\hbar\omega$, introduced in Eq. S7, as a function of *l*. The exact derivative of $\partial n/\partial T$ is,

$$\frac{\partial n}{\partial T}=\frac{\hbar\omega}{k_B T^2}\frac{e^{\hbar\omega/k_B T}}{\left(e^{\hbar\omega/k_B T}-1\right)^2} \qquad \text{S14}$$

which can be normalized by the high temperature approximation $k_B/\hbar\omega$ as

$$\left(\frac{\partial n}{\partial T}\right)\frac{\hbar\omega}{k_B}=\frac{\alpha^2 e^{\alpha}}{\left(e^{\alpha}-1\right)^2} \qquad \text{S15}$$

where $\alpha=\hbar\omega/k_B T$. From equation S8 it is possible to write α as a function of *l* as,

$$\alpha=\frac{\theta_{BZE}}{T}\sqrt{\frac{l_{\min}}{l}} \qquad \text{S16}$$

where $\theta_{BZE}=\hbar\omega_{BZE}/k_B$. In Figure S3 we have plotted $\partial n/\partial T$ normalized by $k_B/\hbar\omega$ as a function of α^{-1}, which is a dimensionless mean free path. At small values of α^{-1}, and hence small values *l* and/or *T*, the high temperature approximation significantly overestimates $\partial n/\partial T$. At larger values of *l* and/or *T* the high temperature approximation is accurate. Points have been placed at the values of l = $l_{\min}$, for *T*=300 K, for all five materials. Since the mean free paths that contribute significantly to thermal conductivity are greater than l_{min}, this represents a worst-case scenario. Hence, at 300 K and 400 K, for these materials, the high temperature approximation assumed to achieve an analytical result in the truncated Debye model is reasonable. We note, however, that this assumption in no way effects the normalization and collapse of the experimental data in Figure 3 of the paper, as the definition of $L_{P,\text{nondimensional}}$ is not dependent on phonon occupation.

Figure S3: Normalized $\partial n/\partial T$ vs. nondimensional phonon mean free path.

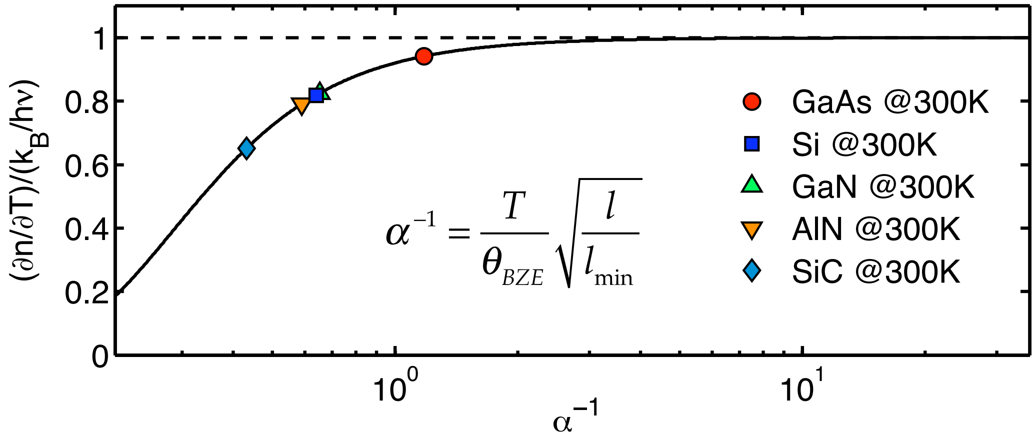

Finally, we note that the phonons measured by BB-FDTR in this work do demonstrate classical occupation. Below is a table showing the difference between the exact expression for $\frac{dn}{dT}$ and the approximation of $\frac{k_B}{\hbar\omega}$ at the minimum measured phonon MFP. Small deviations suggest that it is reasonable to assume that the measured phonons demonstrate classical occupation.

Table S2: Errors from the high temperature approximation of $\partial n/\partial T$

Material	Temperature (K)	Error from High Temp. Approx. at minimum phonon MFP measured (%)	Minimum phonon MFP measured (nm)
GaAs	164	3	380
	330	0.5	262
	445	0.3	191
Si	311	3	376
	417	1	317
GaN	309	4	280
	415	2	241
AlN	308	9	306
	412	2	369
4H-SiC	304	16	339
	407	8	235

6. Normalization parameters for non-dimensionalizing the phonon MFP

To find the values of P, C_U, and $\omega_{BZE} = f_{BZE}2\pi$ that were used to normalize the phonon MFP in Figure 3, bulk thermal conductivity measurements as a function of temperature were fit to

the truncated Debye and Born-von Karman Slack models independently using the relationship, $\Lambda_i = v_g(A\omega^4 + P\omega^2 T e^{-C_U/T} + v_g / b)^{-1}$, where the phonon MFP, Λ_i, is a function of the phonon group velocity, v_g, the material defect scattering rate, *A*, the material dependent constants that define Umklapp scattering rates, *P* and C_U, and the length of the material, *b*. Tables S3 and S4 list the values obtained from the *k* vs. *T* fits for GaAs, Si, GaN, AlN, and 4H-SiC for the truncated Debye and Born-von Karman Slack models, respectively. λ_0 is the smallest allowed wavelength in the Born-von Karman Slack model and is given as $\lambda_0 = 2\pi / (6\pi^2 N)^{1/3}$, where *N* is the number density of primitive cells.

Table S3: Truncated Debye

Material	A (10^{-45} s^3)	P (10^{-19} sK^{-1})	C_U (K)	b (mm)	$f_{BZE,long}$ (10^{12} s^{-1})	$f_{BZE,trans}$ (10^{12} s^{-1})	$v_{s,long}$ (m s^{-1})	$v_{s,trans}$ (m s^{-1})
GaAs	0.5	12.2	81	15 [11]	6.79 [8]	2.35 [8]	4730 [8]	3340 [8]
Si	2.54	3.95	144	130 [12]	12.3 [8]	4.48 [8]	8440 [8]	5850 [8]
GaN	3.5	1.45	173	0.1 [13]	9.0 [9]	6.3 [9]	7960 [9]	4130 [9]
AlN	11.2	1.47	383	3 [14]	10.3 [8]	5.52 [8]	10930 [8]	6200 [8]
4H-SiC	2.20	0.9	237	3.5 [15]	18.0 [10]	8.0 [10]	13100 [10]	7100 [10]

Table S4: Born-von Karman Slack

Material	A (10^{-45} s^3)	P (10^{-19} sK^{-1})	C_U (K)	b (mm)	f_{BZE} (10^{12} s^{-1})	λ_0 (nm)	$v_{s,}$ (m s^{-1}) = $\frac{1}{3}\left(v_{s,long} + 2v_{s,trans}\right)$
GaAs	0.5	4.00	66	15 [11]	4.24	0.55	3803 [8]
Si	2.54	1.53	144	130 [12]	7.77	0.57	6713 [8]
GaN	3.5	0.95	173	0.1 [13]	7.52	0.58	5406 [9]
AlN	7.0	1.20	383	3 [14]	8.86	0.56	7777 [8]
4H-SiC	1.45	0.48	237	3.5 [15]	10.41	0.56	9100 [10]

The model fits and experimental data of thermal conductivity as a function of temperature are shown in Figures S4-S8 for the truncated Debye and Born-von Karman Slack models.

Figure S4:

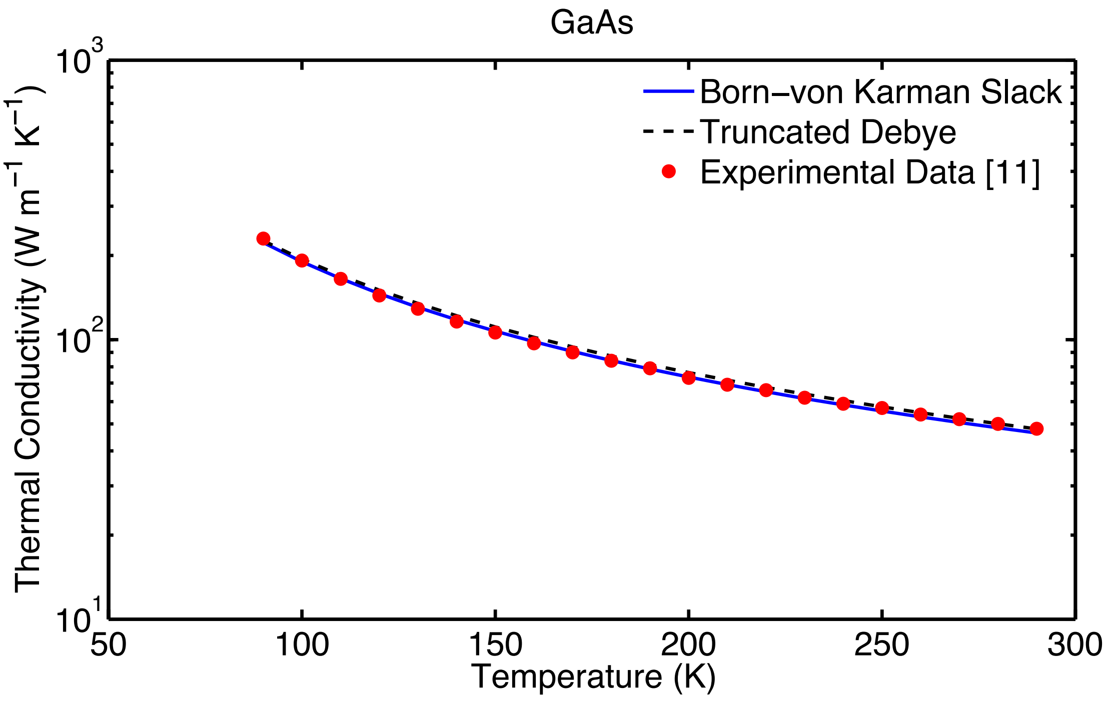

Figure S5:

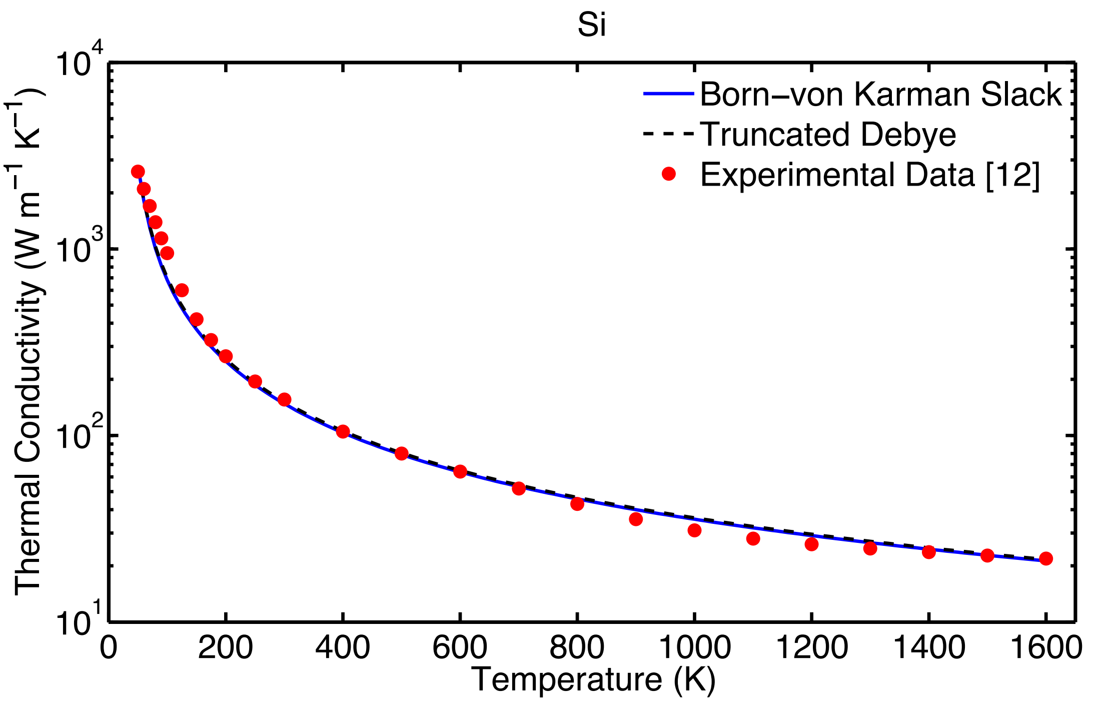

Figure S6:

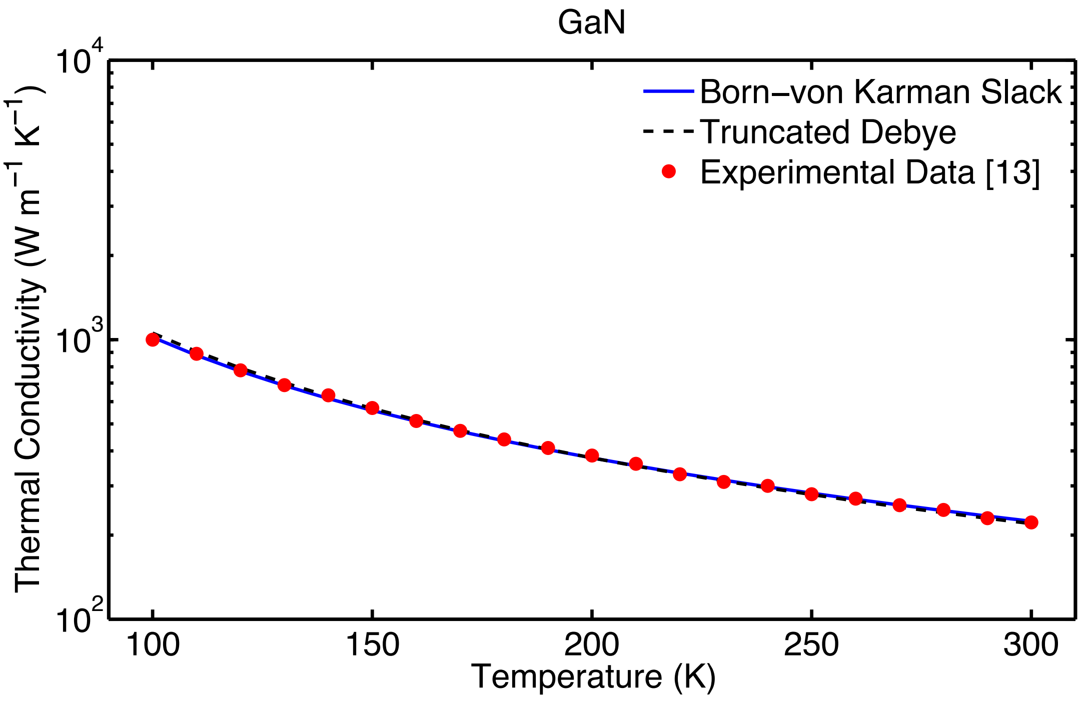

Figure S7:

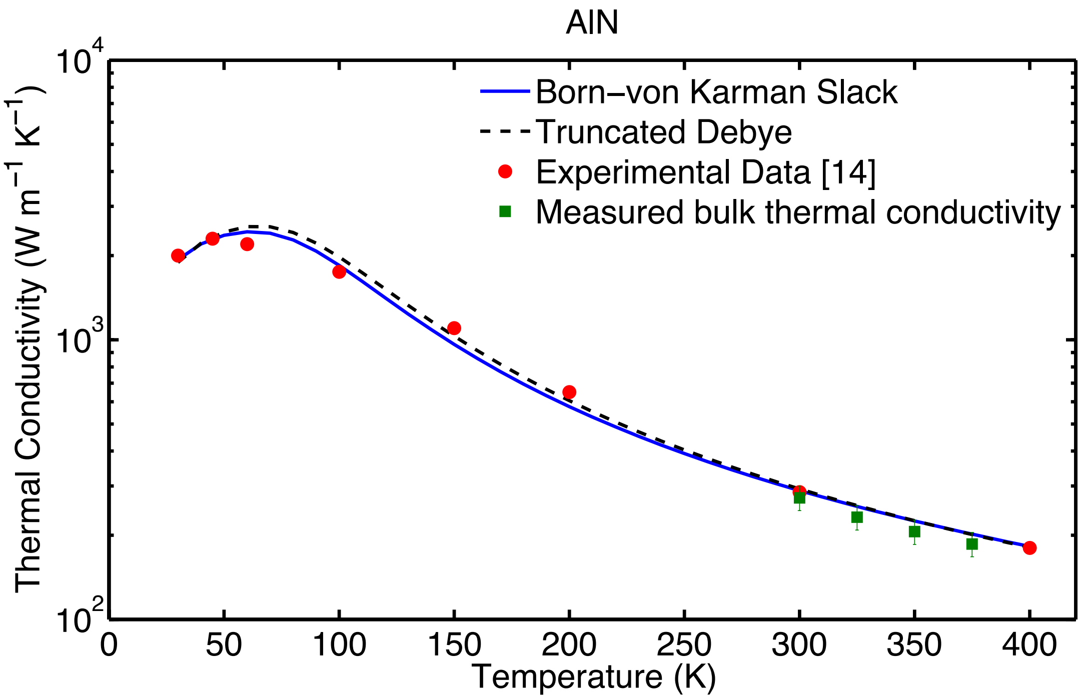

7:

Figure S8:

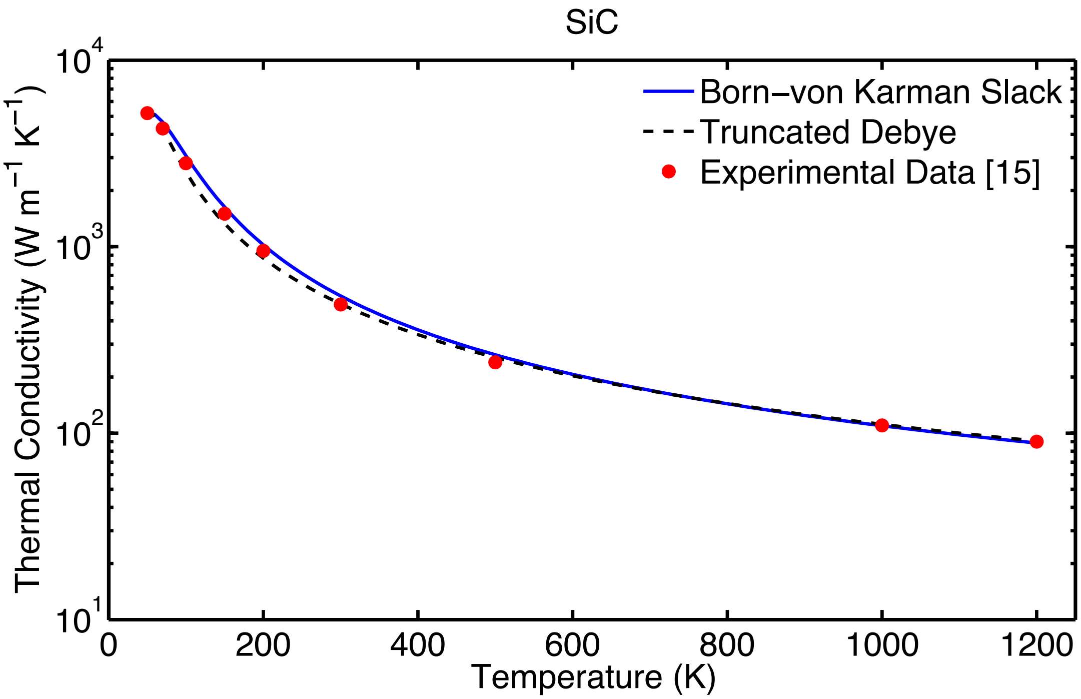

7. Substrate parameters

Table S5:

**Substrate**	**Source**	**Thickness (μm)**	**Resistivity (Ω cm)**
GaAs	University Wafer	625 ± 25	$>10^7$
GaN	Kyma Technologies	475 ± 25	$>10^6$
AlN	Hexatech Inc.	550 ± 50	$>10^{12}$
4H-SiC	Cree Inc.	350 ± 25	$>10^5$

8. Justification of bulk thermal conductivity values

GaAs: The bulk thermal conductivity of GaAs has been documented and has been recently measured [11,16] and predicted by DFT driven simulations [17] over a range of temperatures. Our achievement of full accumulation in GaAs is a good indication of k_{bulk}.

GaN: The GaN substrate used in this study was provided to us by Kyma Technologies and the bulk thermal conductivity of an identical GaN substrate from their growth facility was recently published [18]. Ref. [13] has similar results of bulk thermal conductivity in GaN

near room temperature and was used for low temperature values.

Si: k_{bulk} of Si has a well-known temperature dependence (e.g. from Ref. [19]) given knowledge of the impurity concentration, which is 1×10^{12} cm^{-3} for our sample based on electrical resistivity measurements. Although the phonon mean free path spectra of silicon was not directly measured within this work, since the thermal conductivity accumulation functions of silicon at four different temperatures, normalized by their respective bulk values, are presented as evidence of a universal phonon mean free path spectra, we feel it is necessary to justify the chosen bulk values.

SiC: The 4H-SiC substrate used here was provided to us by Cree Inc. and is designated as high purity semi-insulating (HPSI) within their product specifications [20]. Cree specifies the thermal conductivity of the HPSI 4H-SiC substrates to be 490 W/m-K along the a-axis and 390 W/m-K along the c-axis at room temperature [20]. Since Slack et al.'s [15] a-axis room temperature measurements of 6H-SiC are identical to Cree's measurements of 4H-SiC at room temperature, the values for bulk thermal conductivity of 6H-SiC were used at 80 K, 150 K, and 400 K. Improvement in the thermal conductivity of the 4H-SiC from Cree, relative to measurements from Morelli et al. [21], likely result from improvements in the growth methods of 4H-SiC over the past two decades. A direct quote from Morelli et al. [21] indicates that they suspected stacking faults in the 4H-SiC sample:

> *"In the case of the 4H sample, the phonon mean free path is less than 100 microns [at low temperatures]. This is puzzling since this sample is a single crystal with dimensions on the order of 1mm. The shorter mean free path may by due to the presence of stacking faults in the sample, although we have not done any further experiments to corroborate this claim"*

Even so, deviations between 4H-SiC and 6H-SiC occur primarily at low temperatures in Morelli et al. and it is unlikely that our conclusions of a universal phonon MFP spectrum will be affected. Lastly, Burgemeister et al. [22] states that at room temperature 6H-SiC has a 30% reduced thermal conductivity along the c-axis compared to the a-axis. Cree Inc. [20]

found a similar result, where their 4H-SiC had a 20% reduced thermal conductivity along the c-axis compared to the a-axis at room temperature.

AlN: Unlike the other four materials, our specific AlN samples were not well characterized by recently published values of thermal conductivity. As a spot check in the temperature series, we made 3-omega method [23] measurements of our AlN sample from 300-375K. Our group has familiarity with the 3-omega technique and our measurement setup is identical to that used in Ref. [24], though an insulating layer was unnecessary for the insulating AlN sample. At 300K we found a value of 272 ± 13 W/m-K, which is equivalent to sample W-201 from Ref. [14] (285 ± 14 W/m-K), within the measurement uncertainty. We have plotted our data in Figure S7 over a temperature range 300-375 K for comparison. Given agreement with Ref. [14] within our reported uncertainty (±6%), data from Ref. [14] was used over the extended temperature range.

9. Anisotropic vs. isotropic modeling of 4H-SiC

The figure below shows the k_{accum} data of 4H-SiC at $T = 81$, 151, 304, and 407 K interpreted in two different ways. The anisotropic interpretation [25] assumed a constant ratio of $k_{cross-plane} = 0.8k_{in-plane}$ between the in-plane and cross-plane thermal conductivities of 4H-SiC across all heating frequencies. The isotropic interpretation assumed a ratio of $k_{in-plane} = k_{cross-plane}$ between the in-plane and cross-plane thermal conductivities. At $T = 407$ K and 304 K, the observed thermal conductivity between the anisotropic and isotropic models varied by less than 11% of bulk and less than 4% of bulk at $T = 151$ K and 81 K.

Figure S9: Comparison of Anisotropic to Isotropic Models of k_{accum} in 4H-SiC

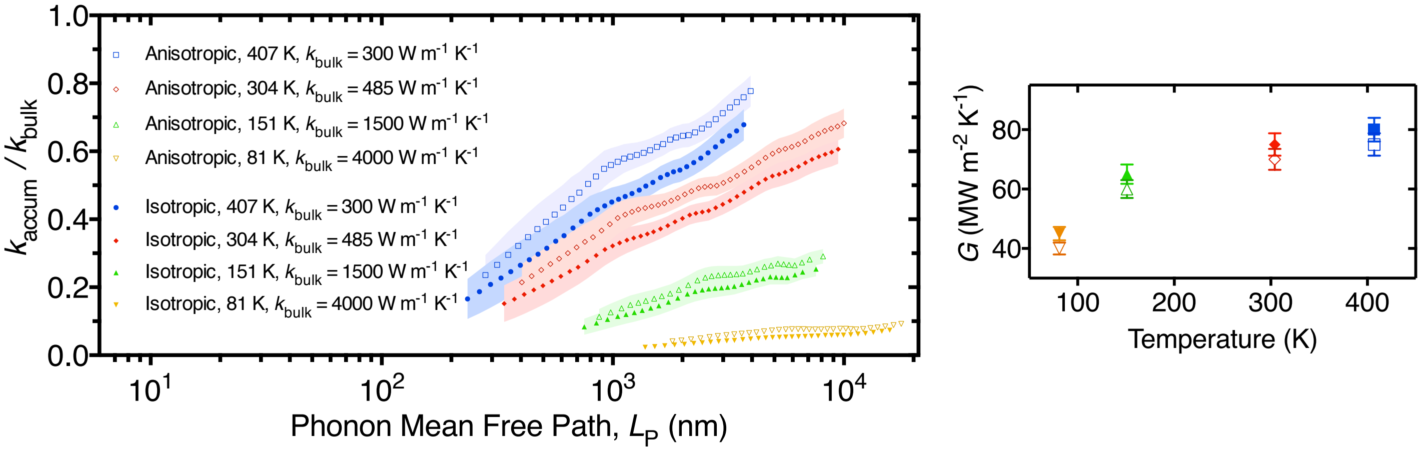

References

[1] J. A. Malen, et al., J. Heat Transfer **133**, 081601 (2011).

[2] K. T. Regner, D. P. Sellan, Z. Su, C. H. Amon, A. J. H. McGaughey, and J. A. Malen, Nat. Commun. **4** 1640 (2013).

[3] K. T. Regner, S. Majumdar, and J. A. Malen, Rev. Sci. Instrum **84**, 064901 (2013).

[4] D. G. Cahill, Rev. Sci. Instrum. **75**, 5119 (2004).

[5] J. S. Blakemore, J. Appl. Phys. **53**, R123 (1982).

[6] M. E. Levinshtein, S. L. Rumyantsev in *Properties of Advanced Semiconductor Materials: GaN, AlN, InN, BN, SiC, SiGe*, edited by M. E. Levinshtein, S. L. Rumyantsev, M. S. Shur (John Wiley & Sons, Inc., New York, USA 2001).

[7] F. Yang and C. Dames, Phys. Rev. B **87**, 035437 (2013).

[8] Y. K. Koh, Ph.D. Thesis, University of Illinois at Urbana-Champaign, 2010.

[9] D. T. Morelli, J. P. Heremans, and G. A. Slack, Phys. Rev. B **66**, 195304 (2002).

[10] D. W. Feldman, J. H. Parker, Jr., W. J. Choyke, and L. Patrick, Phys. Rev. **173** 787-793 (1968).

[11] R. O. Carlson, G. A. Slack, and S. J. Silverman, J. Appl. Phys. **36**, 505 (1965).

[12] C. J. Glassbrenner and G. A. Slack, Phys. Rev. **134**, A1058-A1069 (1964).

[13] A. Jezowski, et al., Solid State Commun. **128**, 69-73 (2003).

[14] G. A. Slack, R. A. Tanzilli, R. O. Pohl, and J. W. Vandersande, J. Phys. Chem. Solids **48**, 641-647 (1987).

[15] G. A. Slack, J. Appl. Phys. **35**, 3460 (1964).

[16] A. V. Inyushkin, et al., Semicond. Sci. Tech. **18**, 685 (2003).

[17] L. Lindsay, D. A. Broido, and T. L. Reinecke, Phys. Rev. B **87** 165201 (2013).

[18] C. Mion, J. F. Muth, E. A. Preble, and D. Hanser, Appl. Phys. Lett. **89**, 092123 (2006).

[19] A. V. Inyushkin, A. N. Taldenkov, A. M. Gibin, A. V. Gusev, and H. J. Pohl, Phys. Status Solidi C **1**, 2995-2998 (2004).

[20] Product Report: *Silicon Carbide Substrates and Epitaxy - Product Specifications*, Cree InC., Durham, NC, USA (2013).

[21] D. T. Morelli, J. P. Hermans, C. Beetz, W. S. Woo, G. L. Harris, C. Taylor, in *Silicon Carbide and Related Materials* Eds. Spencer, M.G., et al., *Institute of Physics Conference Series* N137, 1993, 313-316.

[22] E. A. Burgemeister, W. Vonmuench, and E. Pettenpaul, J. Appl. Phys. **50**, 5790 (1979).

[23] D. G. Cahill, Rev. Sci. Instrum. **61**, 802 (1990).

[24] Z. Su, et al., Appl. Phys. Lett. **100**, 201106 (2012).

[25] A. J. Schmidt, X. Y. Chen, G. Chen, Rev. Sci. Instrum. **79**, 114902 (2008).
